# Controversies in drug allergy: consensus documents from the world experts

**DOI:** 10.1186/s40413-018-0223-2

**Published:** 2018-12-19

**Authors:** Michael Schatz, Alessandro Fiocchi, Erika Jensen-Jarolim, Zuhair K. Ballas

**Affiliations:** 1 Department of Allergy, Kaiser Permanente Medical Center, 7060 Clairemont Mesa Boulevard, San Diego, California 92111 USA; 2 Hospital Bambino Gesù in Rome, Vatican City, Italy; 3 Medical University Vienna and Messerli Research Institute, Vienna, Austria; 4 Univiersity of Iowa and the Iowa City VA, Iowa City, IA USA

**Keywords:** Drug allergy, Hypersensitivity, Beta-lactam, Radiographic contrast media, In vitro allergy testing, Delayed drug reactions

Drug allergy is an extremely important problem worldwide due to its frequency, potential severity, and consequences. It is also a problem that often provides the opportunity for meaningful interaction between allergist-immunologists and other members of the medical community. This includes primary care physicians as well as other medical and surgical specialists, and these interactions may occur not only in outpatient settings but in hospitals and emergency departments as well. The optimal approach to drug allergy often requires a team approach, and allergist-immunologists must be essential members of that team. It is therefore critical that allergist-immunologists provide the best evidence-based recommendations and care possible for this far-reaching condition.

As Editors-in-Chief of the *Journal of Allergy and Clinical Immunology*: *In Practice*, the *World Allergy Organization Journal*, and the *Journal of Allergy and Clinical Immunology*, we have the opportunity to review many articles on the subject of drug hypersensitivity. In that capacity, we have been struck by the wide geographical variation in the approach to the diagnosis and management of a number of types of drug allergy. Although some of this variation may be necessary due to geographical differences in drug use or host responses, we believe that this variation is an opportunity for quality improvement in the care of drug allergy patients around the world. We felt that the Joint Congress of the American Academy of Allergy, Asthma & Immunology and the World Allergy Organization, which was held in March of 2018 in Orlando, Florida, would provide an excellent opportunity for the three journals to sponsor an international consensus conference to try to address some of the most controversial areas in the field of drug allergy. The outcomes of that meeting are included in current issues of our journals. They represent the work of 54 authors from 26 countries and provide a truly global attempt to address the controversies and practice variation in the world of drug allergy.

Published in the *Journal of Allergy and Clinical Immunology*: *In Practice* are articles on the subjects of beta-lactam allergy assessment, from both an individual [1] and an institutional [2] perspective, and hypersensitivity reactions to radiographic contrast media [3]. In the J*ournal of Allergy and Clinical Immunology* are articles on in vitro testing for drug allergy [4] and the approach to delayed drug reactions [5]. In each case, current areas of agreement, areas of disagreement, consensus recommendations, and unmet needs are highlighted. An overall perspective and summary of these articles appears in the *World Allergy Organization Journal* [6].

The editors acknowledge the extraordinary efforts of the co-chairs of this initiative, Mariana Castells and Pascal Demoly. They have skillfully directed all aspects of the project, including topic choices, selection of participants, the organization and running of the in-person meeting, and the coordination and review of the resulting manuscripts. In addition, they have written a superb summary of the various articles and their most important take-away points. This initiative simply would not have been possible without their dedication and inspired leadership. We also express our deep appreciation to all participants and authors who have generously given of their time and expertise without any financial reimbursement. Finally, we thank the *Journal of Allergy and Clinical Immunology: In Practice* Managing Editor, Dawn Angel, for her invaluable logistical support of this endeavor.

We are delighted to have the opportunity to present these extensively researched and thought-out collaborations among the world’s drug allergy experts. We hope these documents will help our clinician readers to provide the most appropriate evidence-based care for the patients they see with drug allergy, including optimal diagnosis, management, and recommendations for future therapy. When options exist, we believe these articles will help practitioners choose the best approach for their particular patients and setting. In addition, we are optimistic that these documents will help allergist-immunologists interact with other members of the health care team to provide optimal drug allergy care on an institutional basis. This series of articles will also help researchers appreciate the unmet needs in the field of drug hypersensitivity and stimulate work as individuals and in collaborations to provide answers to these remaining areas of uncertainty. In this way, we hope these articles will not be an end in themselves but just an important status report toward the ultimate goals of prevention of adverse reactions to drugs to the extent possible and optimizing the quality of care and the quality of life of patients who experience such reactions.
